# Editorial: Smells, Well-Being, and the Built Environment

**DOI:** 10.3389/fpsyg.2022.880701

**Published:** 2022-04-25

**Authors:** Jieling Xiao, Francesco Aletta, Antonella Radicchi

**Affiliations:** ^1^School of Architecture and Design, Birmingham City University, Birmingham, United Kingdom; ^2^Institute for Environmental Design and Engineering, University College London, London, United Kingdom; ^3^Institute of Urban and Regional Planning, Technical University of Berlin, Berlin, Germany

**Keywords:** well-being, smell perception, spatial design and management, environmental planning, tehcnology

## Introduction

**Figure 1:**
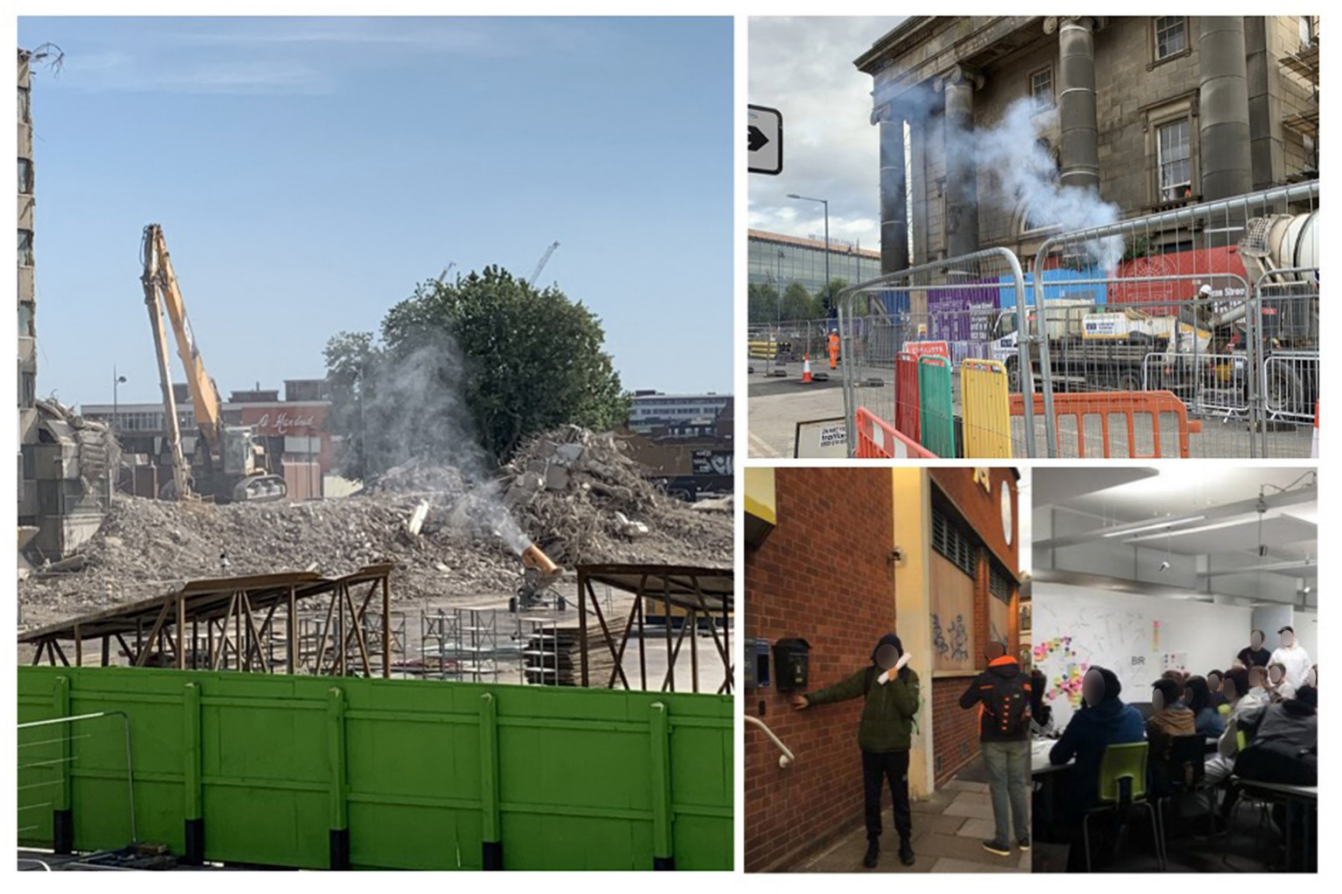
Reading the smells in various sites in the Digbeth area in the city of Birmingham, UK.

From the pungent smells of Khari Baoli Spice Market in New Delhi to soothing smells of Mayfair Lavender Farm in south London, smells bring distinct identities to places and can connect people emotionally to the surroundings (Porteous, 1985). Smells are powerful to influence our feelings and recall memories of the past. Experiences of smells enrich our understanding of places and behavioral responses in places (Classen et al., 1994; Henshaw, 2014; Xiao, 2018) (Figure 1). In light of aromatherapies, spaces with therapeutic smells can potentially bring positive impacts on human wellbeing. In service spaces, smells are important environmental cues to delight people. In artistic practice, smells are curated to create an immersive experience to connect the audience and artists' inner worlds. Conversely smells in the form of odor pollution deriving from waste, traffic, plants, and food districts can compromise the quality of life of residents, and negatively affect our experience of places and lead to behavior changes (Henshaw et al., 2018).

In this Research Topic, we aimed to collect a range of contributions to understand the emotional and wellbeing responses resulting from smells in different public spaces (museums, highstreets, heritage buildings, food districts, neighborhoods, squares, etc.) to inform future spatial design and management. The articles in this Research Topic are presented according to three types of contributions: reviews and conceptual analyses, empirical research in fieldwork, in laboratory studies and technological applications.

## Reviews and Conceptual Analyses

Xiao et al. reviewed smellscape research studies conducted in the past 10 years to identify the challenges and related areas of future research, namely smell archives and databases, social justice within odor control and management, and research into advanced building materials. Spence reviewed the changing role of smells in the built environment from negative associations with sanitation to meaningful personal and cultural associations with memories and experiences which led to an evaluation of different approaches in examining the impact of smells on people's mood or wellbeing and the challenges of researching smells in the multi-sensory environment.

Moving from the sick building sydrome to sick transport sydrome, Spence further reviewed the smells in transport environments as aesthetic and functional, and suggests challenges for future transportation to produce a more tangible vision to integrate smells in the design process to achieve the right balance of olfactory stimulation. Looking backwards to scented past, Bembibre and Strlič make the case for the need of knowledge exchange and interdisciplinary interpretation of findings in the field of olfactory heritage, providing an overview of methodological and museal studies as well as challenges associated with historical scent reconstruction.

## Empirical Research - Fieldwork

Pálsdóttir et al. carried out a field study with participants suffering from stress-related mental disorders and explored how they would describe their smellscape perception of a garden in the context of a nature-based rehabilitation intervention. In a different field study, de Groot investigated whether ambient scents could affect customers' subjective experience and spending behavior in an experiment with customers of a second-hand clothing store. The author concluded that for that to happen, the smellscape should have a meaningful link to the physical context. Masaoka et al. present the results of a study conducted to examine whether continuous odor stimuli associated with autobiographical memories could activate olfactory areas in the brain of older adults and assess whether this odor stimulation could have a protective effect against age-related cognitive decline.

## Empirical Research - Laboratory Studies and Technological Applications

Masaoka et al. investigated the potential protective effect from age-related cognitive decline of continuous odor stimuli associated with autobiographical memories and whether those could activate the above olfactory areas in older adults. Jiang et al. used blood pressure, pulse rate, EEG, POMS, and SD data to examine the odor-visual effects of the *Primula forbesii Franch* compared with the non-fragrant Primula malacoides Franch on the physiological and psychological state of Chinese female college students in the indoor environment. Courrèges et al. examined the correlations between odor and texture in users' perceptions of cosmetic creams cross-culturally, in laboratory conditions, using questionnaires, minimizing the impacts of branded messages from the packing and retail spaces. Amores et al. discussed the design and technical implementation of Essence- a smartphone-controlled wearable device that monitors users' EEG and real-time sleep staging algorithm to release scents to interact with users- in home-based sleep environments.

The articles included in this Research Topic represent a nice balance between the theoretical reviews, empirical studies and laboratory research, showing the vibrance and dynamic in this research field as well as new technological developments such as extended reality, emotional sensors (i.e. EEG, GSR) and odor monitoring devices. New insights are drawn into the theoretical frameworks to understand relationships between smells, wellbeing and emotions, behaviors and physiological aspects; methodological approaches to measure smell triggered emotions, experiences, and quality of life; practical explorations on the process and challenges of using smells to influence user experiences in the built environment.

## Author Contributions

All authors listed have made a substantial, direct, and intellectual contribution to the work and approved it for publication.

## Funding

This editorial project received support via the Conference Network and Mobility Fund from Birmingham City University.

## Conflict of Interest

The authors declare that the research was conducted in the absence of any commercial or financial relationships that could be construed as a potential conflict of interest.

## Publisher's Note

All claims expressed in this article are solely those of the authors and do not necessarily represent those of their affiliated organizations, or those of the publisher, the editors and the reviewers. Any product that may be evaluated in this article, or claim that may be made by its manufacturer, is not guaranteed or endorsed by the publisher.
